# Bilirubin as an indicator of cardiometabolic health: a cross-sectional analysis in the UK Biobank

**DOI:** 10.1186/s12933-022-01484-x

**Published:** 2022-04-18

**Authors:** Nazlisadat Seyed Khoei, Karl-Heinz Wagner, Anja M. Sedlmeier, Marc J. Gunter, Neil Murphy, Heinz Freisling

**Affiliations:** 1 Department of Nutritional Sciences, Faculty of Life Sciences and Research Platform of Active Ageing, University of Vienna, Althanstrasse 14, 1090 Vienna, Austria; 2 Department of Epidemiology and Preventive Medicine, University of Regensburg, Franz-Josef-Strauß-Allee 11, 93053 Regensburg, Germany; 3 Nutrition and Metabolism Branch, International Agency for Research on Cancer (IARC-WHO), 150 Cours Albert Thomas, 69372 Lyon CEDEX 08, France

**Keywords:** Bilirubin, Metabolic syndrome, Obesity, UK Biobank

## Abstract

**Background:**

Mildly elevated bilirubin, a by-product of hemoglobin breakdown, might mitigate cardiometabolic risk factors including adiposity, dyslipidemia, and high blood pressure (BP). We investigated the cross-sectional relationship between (total) bilirubin and baseline cardiometabolic risk factors in 467,519 UK Biobank study participants.

**Methods:**

We used multivariable-adjusted linear regression to estimate associations between bilirubin levels and risk factors of cardiometabolic diseases including body mass index (BMI), waist and hip circumferences (WC, HC), waist-to-hip ratio (WHR), fat mass (FM), and trunk FM, and the blood lipids: apolipoprotein A-I (apoA-I), apolipoprotein B (apoB), apoB/apoA-I, lipoprotein (a), total cholesterol (TC), low-density lipoprotein cholesterol (LDL-C), high-density lipoprotein cholesterol (HDL-C), LDL/HDL, TC/HDL, triglycerides (TG). Log-transformed bilirubin was modelled with restricted cubic splines and predicted mean values with 99% confidence intervals (CI) for each risk marker were estimated, separately. Second, we applied principal component analysis (PCA) for dimension reduction to in turn six anthropometric traits (height, weight, BMI, WC, HC, and WHR) and all above lipids. Last, we estimated associations (99%CI) between bilirubin and three components of the metabolic syndrome, i.e. WC, TG, and BP using logistic regression.

**Results:**

After multivariable adjustments, higher levels of bilirubin were inversely associated with indicators of general adiposity (BMI and FM) and of body fat distribution (WC, HC, WHR, and trunk FM) in both men and women. For example, women with mildly elevated bilirubin (95^th^ percentile equal to 15.0 µmol/L), compared to women with low bilirubin (5^th^ percentile equal to 4.5 µmol/L), had on average a 2.0 kg/m^2^ (99% CI 1.9–2.1) lower BMI. Inverse associations were also observed with dyslipidemia among men and women. For example, mildly elevated bilirubin among men (95^th^ percentile equal to 19.4 µmol/L) compared to low levels of bilirubin (5^th^ percentile equal to 5.5 µmol/L) were associated with approx. 0.55 mmol/L (99% CI 0.53–0.56) lower TG levels, with similar inverse associations among women. Multiple-trait analyses using PCA confirmed single-trait analyses. Men and women with mildly elevated bilirubin levels ≥ 17.1 µmol/L, compared to low-normal bilirubin < 10 µmol/L had 13% (99% CI 8%–18%) and 11% (99% CI 4%–17%) lower odds of exceeding systolic BP levels of ≥ 130 mm Hg, respectively.

**Conclusions:**

Higher levels of bilirubin were inversely associated with cardiometabolic risk factors including adiposity, dyslipidemia, and hypertension.

**Supplementary Information:**

The online version contains supplementary material available at 10.1186/s12933-022-01484-x.

## Introduction

Excess body fatness, hyperlipidemia, and high blood pressure (BP) are among the top seven global risk factors for early death and disability [1], and these three are among the key risk factors for cardiometabolic diseases including type 2 diabetes mellitus (DM) and cardiovascular diseases (CVD) [2, 3]. In contrast, bilirubin, the final product of heme metabolism in the body, is emerging as a cardiometabolic protective agent with preventive and clinical potential [4, 5].

Circulating bilirubin concentrations are primarily derived from hemoglobin of aged or damaged red blood cells and transported to the liver bound to albumin. Serum bilirubin levels are controlled by three enzymes: heme oxygenase (HMOX), biliverdin reductase, and uridine diphosphate glucuronosyltransferase 1A1 (UGT1A1). Within the hepatocytes, UGT1A1 is the only enzyme that contributes to bilirubin glucuronidation, which is essential for the biliary excretion of bilirubin. In humans, there is a benign condition of inheritable hyperbilirubinemia (total bilirubin levels ≥ 17.1 μmol/L) caused by deficiencies of *UGT1A1*, called Gilbert’s syndrome (GS), affecting 5–10% of Caucasians [4].

In an experimental model, mice with hyperbilirubinemia in Gilbert's mutation UGT1A1*28 were resistant against high fat diet-induced adiposity, hyperinsulinemia, and hepatic steatosis [6]. Furthermore, clinical and population based studies suggest that bilirubin plays a role in the pathogenesis of cardiometabolic disorders, including obesity [7–10], which is correlated with systemic inflammation, and accompanied by a pro-oxidative status [11, 12]. Previous research also showed that circulating bilirubin concentrations were negatively correlated with body mass index (BMI), hip circumference (HC), fat mass (FM) [8, 13, 14], and components of the metabolic syndrome (MetS) including abdominal obesity [15–18], triglyceride (TG) levels [19], or BP [20]. However, previous population studies were carried out in specific population groups (e.g. in individuals with GS or in individuals with overweight and obesity), were relatively small (the biggest study to date on this topic included 12,342 participants) limiting the possibility to investigate population sub-groups, and had inconsistent confounder adjustment.

We therefore investigated associations of serum bilirubin concentrations with anthropometric indicators and serum concentrations of blood lipids in the UK Biobank (UKB) with more than 500,000 participants. We selected established and emerging anthropometric and blood lipid risk factors for cardiometabolic diseases as outcomes [21, 22]. These included markers of body fatness (BMI and FM) and body fat distribution (WC, HC, WHR, and trunk FM) as well as dyslipidemia (e.g., TG). We also derived phenotypes of body shape and blood lipid profile and related those to bilirubin concentrations. Lastly, we assessed the odds of having a high waist circumference (WC), elevated TG levels, or high BP as defined by the respective criteria of the MetS (WC > 102 cm in men and > 88 cm in women, TG ≥ 1.7 mmol/L, and BP ≥ 130/85 mm Hg) for clinically defined categories of bilirubin concentrations (low-normal < 10, high-normal 10–17, mildly elevated ≥ 17.1 μmol/L).

## Methods

### Study population

The UKB is a prospective community-based cohort study consisting of over 500,000 participants (54% women) aged 40–69 years, who were recruited between 2006 and 2010 from 22 assessment centers across the UK (England, Wales, and Scotland) with a response rate of 5% [23]. In the present analysis, we report on cross-sectional data at baseline. This research has been conducted using the UKB Resource under application number 55870.

The UKB has approval from the North West Multi-Center Research Ethics Committee, the National Information Governance Board for Health and Social Care in England and Wales, and the Community Health Index Advisory Group in Scotland. In addition, an independent Ethics and Governance Council was formed in 2004 to oversee UKB’s continuous adherence to the Ethics and Governance Framework that was developed for the study (http://www.ukbiobank.ac.uk/ethics/). All participants provided written informed consent.

### Baseline characteristics, anthropometric traits, and body composition

At the baseline assessment, participants reported sociodemographic information, lifestyle habits, and health and medical history factors through touchscreen self- reported questionnaires and nurse-led interviews [24].

Baseline blood samples were collected for measurement of serum biomarkers. Trained research staff measured standing height using the Seca 202 device and body weight using the Tanita BC-418MA [25]. BMI was calculated as weight (in kilograms) divided by square of height (in meters). WHR was derived by dividing WC (in centimeters, measured by a Wessex non-stretchable sprung tape at the level of the umbilicus) by HC (in centimeters, using the same device). Bioelectrical impedance analysis (BIA) was performed using the Tanita BC-418MA Segmental Body Composition Analyzer (Tanita Corporation), which is a single‐frequency (50 kHz) BIA monitor that uses eight polar electrodes and a single‐point load cell weighing system in the scale platform. Measurements were performed on participants in light clothing (after removing of shoes, socks, outer clothing, and contents of pockets). Participants were asked to step on the analyzer, stand straight, and hold the handles while the measurements were taken. It provides separate body mass and impedance readings for different body segments [26, 27]. Exclusions prior to the onset of analyses for this study were participants without a total bilirubin measurement (n = 34,945), resulting in a final sample of 467,519 individuals.

### Blood collection and laboratory methods

As part of the UKB Biomarker Project, all serum parameters were determined by Beckman Coulter AU5800 analyzer (Beckman Coulter (UK), Ltd.). Total bilirubin levels were measured by colorimetric assay, low-density lipoprotein-cholesterol (LDL-C) was analyzed by enzymatic selective protection, high-density lipoprotein-cholesterol (HDL-C) was assessed by enzyme immune-inhibition, apoA-I and apoB were determined by immune-turbidimetric analysis, total cholesterol (TC) and TG were measured by enzymatic kit, and Lp(a) by immuno-turbidimetric method. Liver enzymes (alanine transaminase, ALT, aspartate transaminase, AST, alkaline phosphatase, ALP, and gamma-glutamyl transpeptidase, GGT) were analyzed by enzymatic rate and C-reactive protein (CRP) was assessed by immuno-turbidimetric assay.

For the remainder of this work, bilirubin refers to total bilirubin concentrations.

### Statistical analysis

We decided a priori to run all models separately for men and women because of the well-established sex differences in serum levels of bilirubin [28]. The descriptive characteristics of the study participants were compared across tertiles of bilirubin levels. Data are presented as means and standard deviations (SD), medians and interquartile ranges (IQR), and frequency (percentage) for categorical variables.

We used multivariable-adjusted linear regression analyses to estimate the cross-sectional association between log-transformed bilirubin levels and, separately, each cardiometabolic risk factor adjusted for age at recruitment, ethnicity (White, mixed, Asian, Black, and Chinese), alcohol consumption (never, former, and current), alcohol  consumption frequency (never, special occasions only, 1–3 times/month, 1–2 times/week, 3–4 times/week, and daily or almost daily), smoking status (never, former, and current < 15/day, current 15 + /day, current-intensity unknown), frequency of moderate physical activity (day/week), liver enzyme (alanine transaminase, ALT), chronic diseases (heart problems and DM), medications (for cholesterol, BP, DM, or exogenous hormones), qualifications (none; CSEs/O levels/GCSEs [Certificate of Secondary Education/General Certificate of Secondary Education or equivalent]; vocational qualifications [National Vocational Qualification/Higher National Diploma/Higher National Certificate, A levels/Advanced Subsidiary levels or equivalent]; other qualifications; college/university degree; and unknown), and ever use of hormones in women.

The following single-trait cardiometabolic risk markers were evaluated: the anthropometric measures BMI, WC, HC, WHR, FM, and trunk FM, and the blood lipids apoA-I, apoB, apoB/apoA-I, Lp(a), TC, LDL-C, HDL-C, LDL/HDL, TC/HDL, and TG.

We estimated associations for each outcome by modelling log-transformed bilirubin with restricted cubic splines (3 knots at 10th, 50th, and 90th percentile) allowing for non-linear associations. P-values for non-linearity were computed with log-likelihood ratio tests comparing the spline model to a linear model. P-values < 0.001 were judged as evidence against linearity. Associations are presented graphically as predicted mean values and 99% confidence intervals (CI).

Second, we applied principal component analysis (PCA) to in turn six anthropometric traits (height, weight, BMI, WC, HC, and WHR) in line with Ried et al. [29], and to ten lipid measures (apoA-I, apoB, apoB/apoA-I, Lp (a), TC, LDL-C, HDL-C, TG, LDL/HDL, and TC/HDL). The PCA analysis complements the single-trait analyses to i) account for multicollinearity to potentially identify main drivers of associations and ii) to potentially capture additional phenotype information of complex traits such as body shape and blood lipid profiles that may go undetected in single-trait analyses. For each group, we extracted the first four principal components (PC), which captured most of the variation of the input variables (> 98%). PCA was performed on the standardized residuals of the anthropometric traits adjusted for age at recruitment, sex, and study center. The same analyses were repeated for lipid measures. The degree of correlation between variables and PCs are given by variable loadings, and indicates higher influence of a given variable on a PC [30, 31].

We repeated the multivariable-adjusted linear regression analysis with log-transformed bilirubin modelled with restricted cubic splines as described above, where the anthropometric measures were replaced with the corresponding four mutually adjusted PCs for anthropometric and in turn with the four mutually adjusted PCs for blood lipid measures. To better illustrate each body shape, the mean values of the top and bottom 5% proportions of each individual were calculated for each PC and plotted using the online program https://bodyvisualizer.com/.

Last, we assessed the association between bilirubin and three components of the MetS, i.e. WC, TG, and BP using logistic regression analyses. For this analysis we applied clinically relevant cut-off values for bilirubin (low-normal < 10, high-normal 10–17, mildly elevated ≥ 17.1 μmol/L) [32, 33] and adopted the NCEP ATP III proposed criteria to dichotomize the three selected components of the MetS: 1) WC > 102 cm in men and > 88 cm in women; 2) TG ≥ 1.7 mmol/L; and 3) BP ≥ 130/85 mm Hg [34]. Each of the three models were adjusted for all confounders mentioned above and linearity was evaluated with a linear contrast across tertiles.

We performed sensitivity analyses excluding participants with prevalent cardiometabolic conditions (heart attack, angina, stroke, and DM). Second, we further adjusted our main model for CRP to evaluate the role of inflammation in observed associations. Third, we assessed the influence of high levels of circulating liver enzymes on our results, which could indicate sub-clinical liver dysfunction and thereby increasing bilirubin levels, by excluding participants in the highest decile of circulating ALT, AST, ALP, and GGT.

Analyses were conducted in Stata version 16.1 (Stata Corp, College Station, TX, USA). Statistical tests were two-sided and a p-value < 0.001 after Bonferroni correction (0.05 over 38 tests) was considered statistically significant and corresponding 99% CI were estimated.

## Results

### General characteristics of the study population

Table 1 describes the main characteristics of the study population by tertiles of bilirubin. Compared to the lowest tertile of bilirubin, men and women in the highest tertile were taller and slimmer (lower weight, BMI, WC, and FM), had lower TC, LDL-C, Lp(a), LDL/HDL, TC/HDL, TG, and higher HDL-C, were more likely to have a college/university degree and were less likely to be current smokers. Women in the highest tertile were also more likely to consume alcohol as compared to women in the lowest tertile.

**Table 1:** Characteristics of UK Biobank study participants by category of circulating total bilirubin levels (N = 467,519)

Baseline characteristic	Total bilirubin tertile			Total bilirubin tertile		
	1	2	3	1	2	3
	Men			Women		
	n = 71,496	n = 71,270	n = 71,240	n = 84,930	n = 84,185	n = 84,398
Bilirubin levels (μmol/L)^Ω^	6.7 [5.9; 7.4]	9.1 [8.5; 9.8]	13.2 [11.7; 16.5]	5.4 [4.8; 5.9]	7.3 [6.8; 7.8]	10.4 [9.2; 12.8]
Age (years)^a^	56.6 (8.2)	56.9 (8.1)	56.8 (8.3)	56.3 (7.9)	56.8 (7.9)	56.0 (8.2)
Weight (kg)^a^	86.3 (15.1)	86.0 (14.1)	85.5 (13.7)	73.0 (14.7)	71.3 (13.9)	69.9 (13.3)
Height (cm)^a^	174.9 (6.8)	175.8 (6.8)	176.2 (6.9)	161.8 (6.3)	162.5 (6.3)	163.1 (6.3)
Body mass index (kg/m^2^)^a^	28.2 (4.5)	27.8 (4.1)	27.5 (4.0)	27.9 (5.4)	27.0 (5.1)	26.3 (4.9)
Waist circumference (cm)^a^	97.9 (11.8)	96.8 (11.1)	96.0 (10.9)	86.7 (12.9)	84.5 (12.3)	82.8 (12.0)
Hip circumference (cm)^a^	103.6 (8.1)	103.4 (7.5)	103.2 (7.2)	104.6 (10.9)	103.3 (10.2)	102.1 (9.8)
Waist-hip ratio^a^	0.9 (0.1)	0.9 (0.1)	0.9 (0.1)	0.8 (0.1)	0.8 (0.1)	0.8 (0.1)
Fat mass (kg)^a^	22.8 (8.7)	22.3 (8.1)	21.8 (7.8)	28.3 (10.4)	26.9 (9.9)	25.6 (9.6)
Trunk fat mass (kg)^a^	14.1 (5.2)	13.8 (5.0)	13.5 (4.9)	14.3 (5.4)	13.6 (5.2)	13.0 (5.1)
Apolipoprotein A (g/L)^a^	1.4 (0.2)	1.4 (0.2)	1.4 (0.2)	1.6 (0.3)	1.6 (0.3)	1.7 (0.3)
Apolipoprotein B (g/L)^a^	1.0 (0.2)	1.0 (0.2)	1.0 (0.2)	1.0 (0.2)	1.0 (0.2)	1.0 (0.2)
Apolipoprotein B/Apolipoprotein A-I^a^	0.8 (0.2)	0.7 (0.2)	0.7 (0.2)	0.7 (0.2)	0.7 (0.2)	0.6 (0.2)
Lipoprotein (a) (nmol/L)^a^	44.1 (49.3)	44.0 (49.0)	43.7 (48.8)	45.9 (49.8)	45.3 (49.4)	44.6 (48.8)
Total cholesterol (mmol/L)^a^	5.5 (1.1)	5.5 (1.1)	5.4 (1.1)	5.9 (1.1)	5.9 (1.1)	5.8 (1.1)
Low-density lipoprotein cholestrol (mmol/L)^a^	3.5 (0.9)	3.5 (0.9)	3.4 (0.9)	3.6 (0.9)	3.7 (0.9)	3.6 (0.9)
High-density lipoprotein cholestrol (mmol/L)^a^	1.2 (0.3)	1.3 (0.3)	1.3 (0.3)	1.5 (0.4)	1.6 (0.4)	1.6 (0.4)
LDL/HDL^a^	2.9 (0.9)	2.9 (0.9)	2.8 (0.9)	2.5 (0.8)	2.4 (0.8)	2.3 (0.8)
TC/HDL^a^	4.6 (1.2)	4.5 (1.1)	4.3 (1.1)	4.0 (1.0)	3.8 (1.0)	3.7 (1.0)
Triglycerides (mmol/L)^a^	2.2 (1.2)	1.9 (1.1)	1.8 (1.0)	1.8 (1.0)	1.5 (0.8)	1.4 (0.8)
C-reactive protein (mg/L)^a^	3.0 (5.0)	2.3 (4.1)	2.1 (3.9)	3.4 (5.0)	2.6 (4.1)	2.1 (3.7)
Systolic blood pressure (mm Hg)^a^	143.0 (18.4)	142.8 (18.5)	142.3 (18.6)	137.5 (19.9)	137.5 (20.3)	136.5 (20.6)
Diastolic blood pressure (mm Hg)^a^	83.8 (10.5)	84.1 (10.5)	84.1 (10.6)	80.8 (10.5)	80.7 (10.5)	80.5 (10.6)
Duration of moderate activity (min/d)^a^	66.0 (88.2)	63.8 (83.1)	61.3 (79.2)	55.1 (69.1)	56.3 (68.6)	55.8 (68.5)
Ever used hormone-replacement therapy^b^						
Yes				40	39	35
Qualification^b^						
College/uni degree	30	35	36	28	31	34
Smoking status^b^						
Never	43	50	54	57	60	62
Former	38	39	38	30	32	32
Current	18	11	8	12	8	6
Alcohol consumption status^b^						
Never	3	3	3	7	6	5
Former	4	3	3	5	3	3
Current	92	94	94	88	91	92

Data are presented as Ω median [interquartile range (25^th^ percentile and the 75^th^ percentile)] for non-normal distributed measures, ^a^mean (SD) for continuous measures, ^b^ n% for categorical measures

Number of missing data in main outcomes (n): weight (1557), height (1360), waist circumference (999), hip circumference (1054), fat mass (9080), trunk fat mass (8618), apolipoprotein A-I (41,982), apolipoprotein B (2319), lipoprotein (a) (93,429), total cholesterol (140), low-density lipoprotein cholestrol (841), high-density lipoprotein cholestrol (39,571), triglycerides (174), systolic blood pressure (30,099), diastolic blood pressure (30,087)

### Bilirubin and associations with measures of anthropometry and blood lipids

**Fig. 1:**
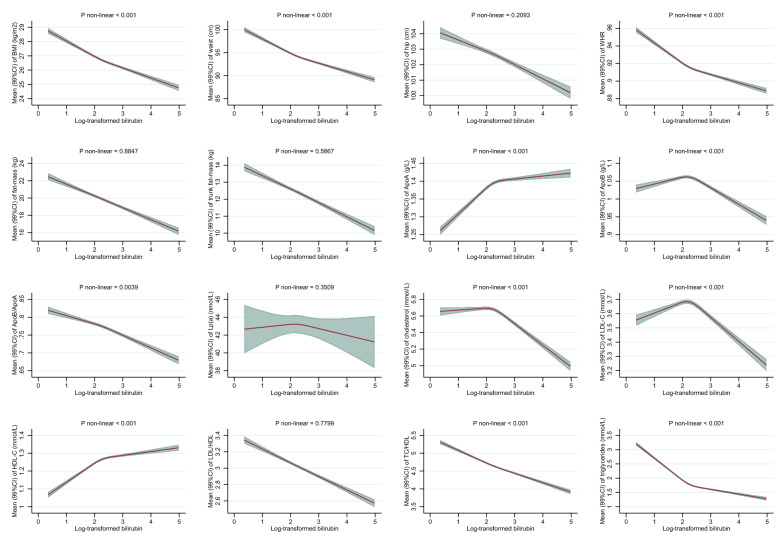

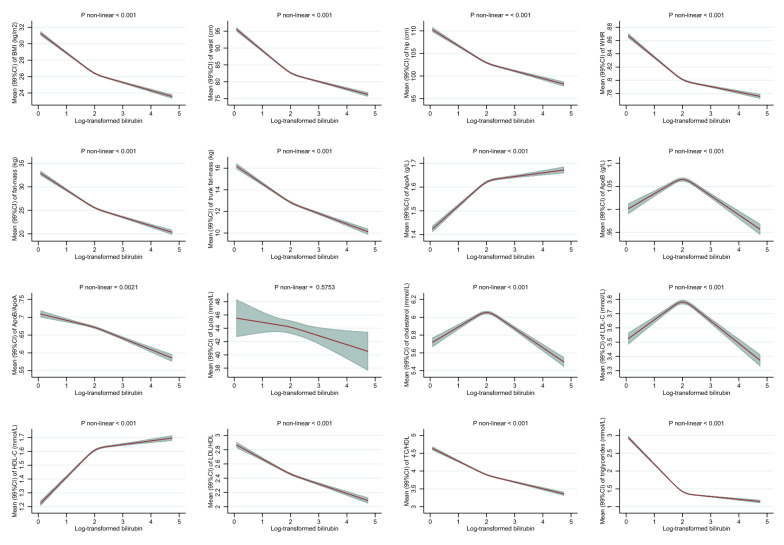
**A** Associations between total bilirubin concentrations and anthropometric measures and lipid profile among men in the UK Biobank. BMI: body mass index, WHR: waist-hip ratio, ApoA-I: apolipoprotein A-I, ApoB: apolipoprotein B, Lp (a): lipoprotein (a), LDL-C: low-density lipoprotein cholesterol, HDL-C: high-density lipoprotein cholesterol. Associations were estimated for each outcome by modelling log-transformed bilirubin with restricted cubic splines (3 knots at 10th, 50th, and 90th percentile) allowing for non-linear associations (adjusted for age at recruitment, ethnicity, alcohol consumption, alcohol consumption frequency, smoking status, physical activity, liver enzyme (alanine transaminase), chronic diseases (heart problems and diabetes), medications (for cholesterol, blood pressure, diabetes, or exogenous hormones), qualifications, and ever use of hormones among women). P-values for non-linearity were computed with log-likelihood ratio tests comparing the spline model to a linear model. P-values < 0.001 were judged as evidence against linearity. Associations are presented as predicted mean values and 99% confidence intervals (CI). Levels of (total) bilirubin among men at 5^th^, 10^th^ (1^st^ knot), 50^th^ (2^nd^ knot), 90^th^ (3^rd^ knot), and 95^th^ were 5.45, 6.08, 9.13, 15.51, and 19.42 on original scale (µmol/L) and 1.70, 1.81, 2.21, 2.74, and 2.97 on log-scale, respectively. **B** Associations between total bilirubin concentrations and anthropometric measures and blood lipid concentrations among women in the UK Biobank. BMI: body mass index, WHR: waist-hip ratio, ApoA-I: apolipoprotein A-I, ApoB: apolipoprotein B, Lp (a): lipoprotein (a), LDL-C: low-density lipoprotein cholesterol, HDL-C: high-density lipoprotein cholesterol. Associations were estimated for each outcome by modelling log-transformed bilirubin with restricted cubic splines (3 knots at 10^th^, 50th, and 90th percentile) allowing for non-linear associations (adjusted for age at recruitment, ethnicity, alcohol consumption, alcohol consumption frequency, smoking status, physical activity, liver enzyme (alanine transaminase), chronic diseases (heart problems and diabetes), medications (for cholesterol, blood pressure, diabetes, or exogenous hormones), qualifications, and ever use of hormones among women). P-values for non-linearity were computed with log-likelihood ratio tests comparing the spline model to a linear model. P-values < 0.001 were judged as evidence against linearity. Associations are presented as predicted mean values and 99% confidence intervals (CI). Levels of (total) bilirubin among women at 5^th^, 10^th^ (1^st^ knot), 50^th^ (2^nd^ knot), 90^th^ (3^rd^ knot), and 95^th^ were 4.46, 4.94, 7.26, 12.07, and 15.02 on original scale (µmol/L) and 1.50, 1.60, 1.98, 2.49, and 2.71 on log-scale, respectively

After multivariable adjustments, higher levels of bilirubin were associated with favorable anthropometric traits among men (Fig. 1A) and similarly among women (Fig. 1B). The strongest inverse association was observed for BMI among women, where women with mildly elevated bilirubin (95^th^ percentile equal to 15.0 µmol/L), compared to women with low levels of bilirubin (5^th^ percentile equal to 4.5 µmol/L), had on average a 2.0 kg/m^2^ (99% CI 1.9–2.1) lower BMI (26 vs 28 kg/m^2^) (Fig. 1B). Associations with FM, also indicating general adiposity, and indicators of body fat distribution (WC, HC, WHR, and trunk FM) were similarly inverse in both men and women. Although non-linearity was detected in 13 out of 16 associations (P < 0.001), spline plots showed only minor departure from linearity (Fig. 1A and 1B).

Higher levels of bilirubin were also associated with favorable blood lipid concentrations, similarly among men (Fig. 1A) and women (Fig. 1B). For example, mildly elevated bilirubin among men (95^th^ percentile equal to 19.4 µmol/L) compared to low levels of bilirubin (5^th^ percentile equal to 5.5 µmol/L) were associated with approx. 0.55 mmol/L (99% CI 0.53–0.56) lower TG levels, with similar inverse associations among women. In contrast, positive associations were observed with HDL-C as well as apoA-I among men and women, respectively. These observed associations were generally steeper for bilirubin increments from low levels to median levels and tended to flatten off at higher levels (Fig. 1A and B). Inverse U-shaped associations were observed for apoB, TC, and LDL-C, where associations were close to null for low bilirubin up to median bilirubin levels, and inverse for bilirubin levels above the median. No marked associations were observed between bilirubin and Lp(a) (Fig. 1A and B).

### PC patterns of anthropometric measures and blood lipid concentrations

The extracted four PCs for both anthropometric measures and blood lipid concentrations explained more than 98% of the total variation. The loading coefficients of each PC are shown in Additional file 1: Table S1A (anthropometry) and S1B (blood lipids). PC1_anthropometry_ had equally high positive loadings on all anthropometric traits, except for height, characterizing individuals with general adiposity. PC2_anthropometry_ had high but opposite loadings on height and WHR, capturing variation in a composite body shape that represents tall individuals with a low WHR or vice versa. PC3_anthropometry_ had high positive loadings on height and WHR, and an opposite loading of nearly the same size on HC, characterizing tall individuals with a high WHR resulting from a smaller HC or vice versa. PC4_anthropometry_ could be interpreted as a phenotype ranging between high BMI and weight, with relatively small HC and WC vs. low BMI and weight but large HC and WC [29]. Additional file 1: Figure S1 shows these body shapes graphically (95^th^ percentile of each body shape).

PC1_lipids_ characterizes individuals with an overall unfavorable blood lipid profile with high loadings on all blood lipids, except apoA-I and HDL-C, both of which had loadings in the opposite direction, and a negligible loading of Lp(a). PC2_lipids_ characterizes individuals with a favorable blood lipid profile, which could be described as anti-atherogenic, exhibiting virtually a reversed blood lipid pattern to PC1_lipids_. PC3_lipids_ contrasts individuals with high vs low circulating levels of Lp(a), while all other lipids contributed very little to this lipid pattern. PC4_lipids_ had very high loadings on TG and moderately high loadings on apoA-I and Lp(a).

### Bilirubin and associations with body shape phenotypes

**Fig. 2:**
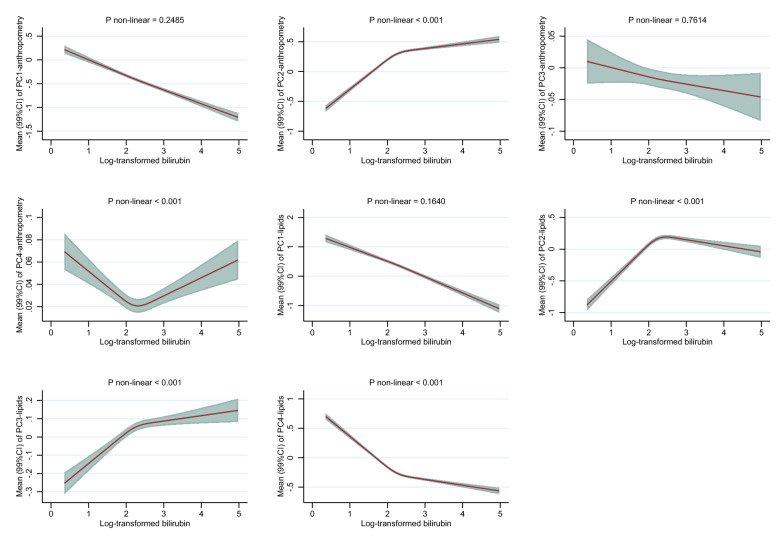

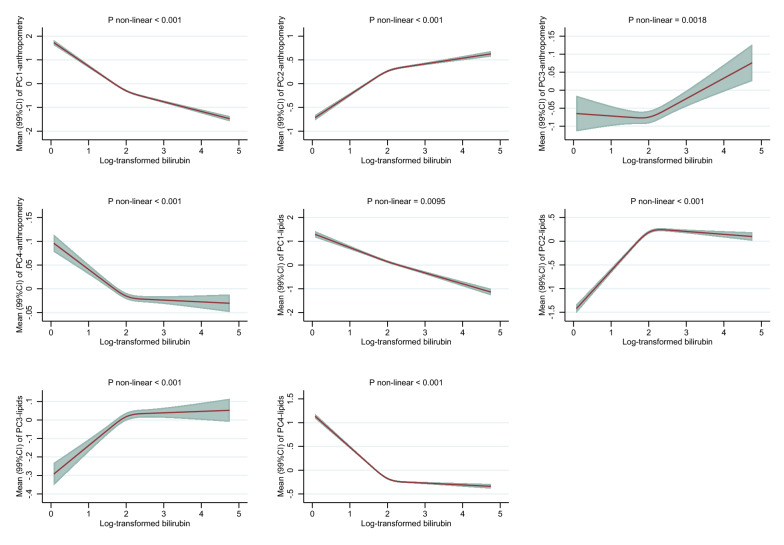
**A**. Associations between total bilirubin concentrations and PC_anthropometry_ and PC_lipids_ among men in the UK Biobank. BMI: body mass index, WHR: waist-hip ratio, ApoA-I: apolipoprotein A, ApoB: apolipoprotein B, Lp (a): lipoprotein (a), LDL-C: low-density lipoprotein cholesterol, HDL-C: high-density lipoprotein cholesterol. PC1_anthropometry_: General adiposity, PC2_anthropometry_: Tall with low waist-to-hip ratio, PC3_anthropometry_: Tall with high waist-to-hip ratio, PC4_anthropometry_: High BMI and weight, with relatively small hip and waist circumference. PC1_lipids:_ Dyslipidemia, PC2_lipids:_ Anti-atherogenic, PC3_lipids:_ High levels of lipoprotein (a), PC4_lipids:_ High levels of triglycerides. Associations were estimated for each outcome by modelling log-transformed bilirubin with restricted cubic splines (3 knots at 10^th^, 50^th^, and 90^th^ percentile) allowing for non-linear associations (adjusted for age at recruitment, ethnicity, alcohol consumption, alcohol consumption frequency, smoking status, physical activity, liver enzyme (alanine transaminase), chronic diseases (heart problems and diabetes), medications (for cholesterol, blood pressure, diabetes, or exogenous hormones), qualifications, and ever use of hormones among women). P-values for non-linearity were computed with log-likelihood ratio tests comparing the spline model to a linear model. P-values < 0.001 were judged as evidence against linearity. Associations are presented as predicted mean values and 99% confidence intervals (CI). Levels of (total) bilirubin among men at 5^th^, 10^th^ (1^st^ knot), 50^th^ (2^nd^ knot), 90^th^ (3^rd^ knot), and 95^th^ were 5.45, 6.08, 9.13, 15.51, and 19.42 on original scale (umol/L) and 1.70, 1.81, 2.21, 2.74, and 2.97 on log-scale, respectively. **B** Associations between total bilirubin concentrations and PC_anthropometry_ and PC_lipids_ concentrations among women in the UK Biobank. BMI: body mass index, WHR: waist-hip ratio, ApoA-I: apolipoprotein A, ApoB: apolipoprotein B, Lp (a): lipoprotein (a), LDL-C: low-density lipoprotein cholesterol, HDL-C: high-density lipoprotein cholesterol. PC1_anthropometry_: General adiposity, PC2_anthropometry_: Tall with low waist-to-hip ratio, PC3_anthropometry_: Tall with high waist-to-hip ratio, PC4_anthropometry_: High BMI and weight, with relatively small hip and waist circumference. PC1_lipids:_ Dyslipidemia, PC2_lipids:_ Anti-atherogenic, PC3_lipids:_ High levels of lipoprotein (a), PC4_lipids:_ High levels of triglycerides. Associations were estimated for each outcome by modelling log-transformed bilirubin with restricted cubic splines (3 knots at 10^th^, 50^th^, and 90^th^ percentile) allowing for non-linear associations (adjusted for age at recruitment, ethnicity, alcohol consumption, alcohol consumption frequency, smoking status, physical activity, liver enzyme (alanine transaminase), chronic diseases (heart problems and diabetes), medications (for cholesterol, blood pressure, diabetes, or exogenous hormones), qualifications, and ever use of hormones among women). P-values for non-linearity were computed with log-likelihood ratio tests comparing the spline model to a linear model. P-values < 0.001 were judged as evidence against linearity. Associations are presented as predicted mean values and 99% confidence intervals (CI). Levels of (total) bilirubin among women at 5^th^, 10^th^ (1st knot), 50^th^ (2nd knot), 90^th^ (3rd knot), and 95^th^ were 4.46, 4.94, 7.26, 12.07, and 15.02 on original scale (umol/L) and 1.50, 1.60, 1.98, 2.49, and 2.71 on log-scale, respectively

After multivariable adjustments, bilirubin was inversely associated with PC1_anthropometry_ among men and women (Fig. 2A and 2B). There was a -0.4 SD-unit difference in PC1_anthropometry_ comparing men with mildly elevated bilirubin (95^th^percentile equal to 19.4 µmol/L) to low levels of bilirubin (5^th^ percentile equal to 5.5 µmol/L). Corresponding associations among women were similarly inverse (Fig. 2B). Positive associations, among men and women, were observed for PC2_anthropometry_. Among men, bilirubin was inversely associated with PC3_anthropometry_, but positively associated among women (Fig. 2B). Among women, but not men, bilirubin tended to be inversely associated with PC4_anthropometry_.

### Bilirubin and associations with blood lipid patterns

Bilirubin was linearly inversely associated with PC1_lipids_, similarly among men and women. Non-linear positive associations were observed with PC2_lipids_, exhibiting a steep increase from low to median levels of bilirubin and a null association from median to higher levels of bilirubin. Similar non-linear positive associations were observed with PC3_lipids_. Bilirubin was non-linearly inversely associated with PC4_lipids_. These associations were most pronounced between low to median levels of bilirubin, among men and women, and flattened off between median to elevated levels of bilirubin (Fig. 2A and B).

### Bilirubin and associations with components of the MetS

Men and women with bilirubin levels ≥ 17.1 µmol/L, compared to bilirubin < 10 µmol/L had 32% (99% CI 29%-34%) and 46% (99% CI 43%-49%) lower odds of exceeding the clinical cut off for WC (> 102 cm in men and > 88 cm in women), respectively. Similar inverse associations were observed for exceeding the upper clinical cut off for TG (Table 2). Furthermore, men and women with bilirubin levels ≥ 17.1 µmol/L, compared to bilirubin < 10 µmol/L had 13% (99% CI 10%–17%) and 11% (99% CI 6%–15%) lower odds of exceeding systolic BP levels of ≥ 130 mm Hg (i.e. hypertension), respectively.

**Table 2:** Odds ratios and 99% confidence intervals for association between three components of the metabolic syndrome and clinical bilirubin levels

Bilirubin cut-off (μmol/L)		N (men/women)	Men		Women	
			OR (99% CI)	*P trend*	OR (99% CI)	*P trend*
Adjusted models						
WC						
Ref.	< 10	128,981/205,724	–	< 0.001	–	< 0.001
1	10–17	68,710/39,020	0.80 (0.78 to 0.83)		0.73 (0.70 to 0.75)	
2	≥ 17.1	15,862/8,223	0.68 (0.65 to 0.72)		0.54 (0.50 to 0.58)	
TG						
Ref	< 10	129,224/206,137	–	< 0.001	–	< 0.001
1	10–17	68,802/39,078	0.65 (0.63 to 0.66)		0.62 (0.59 to 0.64)	
2	≥ 17.1	15,874/8,230	0.58 (0.55 to 0.60)		0.52 (0.48 to 0.56)	
SBP						
Ref.	< 10	121,051/192,488	–	< 0.001	–	< 0.001
1	10–17	64,434/36,713	0.90 (0.88 to 0.93)		0.91 (0.88 to 0.94)	
2	≥ 17.1	14,965/7,769	0.87 (0.82 to 0.92)		0.89 (0.83 to 0.96)	
DBP						
Ref.	< 10	121,052/192,493	–	< 0.001	–	< 0.001
1	10–17	64,439/36,714	1.00 (0.98 to 1.03)		0.95 (0.92 to 0.99)	
2	≥ 17.1	14,965/7,769	0.94 (0.89 to 0.98)		0.88 (0.83 to 0.95)	

Logistic regression model adjusted for age at recruitment, ethnicity, alcohol consumption, alcohol consumption frequency, smoking status, physical activity, liver enzyme (alanine transaminase), chronic diseases (heart problems and diabetes), medications (for cholesterol, blood pressure, diabetes, or exogenous hormones), qualifications, and ever use of hormones among women

WC, waist circumference; TG, triglycerides; SBP, systolic blood pressure; DBP, diastolic blood pressure; OR, odds ratio; CI, confidence interval

The clinical cut-off of these three components of the metabolic syndrome were: (1) WC > 102 cm in men and > 88 cm in women; (2) TG ≥ 1.7 mmol/L; (3) BP ≥ 130/85 mm Hg

*P-trend* computed as linear contrast

### Sensitivity analyses

Sensitivity analyses showed that excluding participants with CVD (n = 26,956) and/or T2D (n = 24,395) at baseline or participants in the highest decile of circulating levels of ALT (n = 222), AST (n = 29), ALP (n = 109), and GGT (n = 234) did not alter observed associations (see Additional file 1: Table S2A–B, model S1, S2, and S3, respectively). Further adjustment of body shape PCs for CRP led to some attenuation of observed associations between bilirubin and PC1 (general adiposity), particularly among women, and PC3, only among women (see Additional file 1: Table S2A, model S4).

## Discussion

In this cross-sectional analysis, we found that higher circulating levels of bilirubin were inversely associated with cardiometabolic risk factors (several indicators of adiposity, blood lipid concentrations, and blood pressure) among men and women in the UKB cohort. PCA analyses that accounted for the correlation among investigated risk factors confirmed findings of our single-trait analysis. Individuals with mildly elevated bilirubin levels indicative of GS (≥ 17.1 µmol/L) were also less likely to exceed the clinical thresholds for the three investigated components of the MetS; i.e. WC > 102 cm among men and > 88 cm among women, TG levels ≥ 1.7 mmol/L, and systolic BP ≥ 130 mm Hg.

Alterations in plasma lipids and lipoprotein particles can promote CVD  [42]. GS individuals have lower serum TC, LDL-C, and oxidized LDL (ox-LDL), and TG [7–9, 35, 36]. A similar profile was observed in both humanized mice with Gilbert's mutation as well as diet-induced obese mice treated with unconjugated bilirubin [6, 37]. The degree to which bilirubin signaling impacts these effects on serum lipids is not known; however, as a selective ligand for peroxisome proliferator activated receptor alpha (PPARα) agonists (like hypolipidemic fibrate), bilirubin could favorably modulate lipid oxidation and inflammation markers [4, 38–40]. Similar to our results, other cross-sectional [13] or case–control studies [7, 8] showed inverse relationships between bilirubin levels and abdominal obesity, insulin resistance, fasting TG, TC, and LDL-C. The same negative association, as expected, was seen for apoB [41], which is also an independent predictor of atherosclerotic disease [42]. In contrast, there appears to be a positive association between bilirubin and a blood lipid pattern driven by Lp(a)–PC3_lipids_ (Fig. 2A and B). This was a surprising finding since we rather expected an inverse association given that Lp(a) is thought to be a risk factor for CVD. However, it is also believed that high plasma Lp(a) levels are not causally associated with CVD risk [1]. Instead, it is thought to confer additional risk only in the presence of traditional risk factors [2]. If the latter were true, then PC3_lipids_ would probably not be indicative of a CVD-risk increasing blood lipid pattern, because all other blood lipids contributed very little to this PC. There is almost no literature linking bilirubin and Lp(a) [43]. Congruent with our results of Lp(a) that did not account for other blood lipids, Nwose et al. reported a very weak inverse association between bilirubin and Lp(a) (r = − 0.016) [43]. If anything, they argue that oxidative stress could be a possible link between these two molecules, whereby oxidative stress could induce hemolysis and in turn hyper-bilirubinemia, and Lp(a) could be independently elevated due to oxidative stress [43]. These arguments either suggest a non-causal spurious association or a complex interaction, which requires further study. The direct relationship between bilirubin and CVD is still debated [44, 45], suggesting a complex relationship. We argue that our findings will help to clarify this relationship by providing evidence for potential pathways between bilirubin and cardiometabolic diseases, for example, through specific blood lipids or blood lipid patterns.

Since weight reduction is known to reduce several cardiometabolic risk factors, it is important to note that short-term (4 weeks) weight-loss combined intervention with administration of sibutramine, diet, and physical activity could increase bilirubin concentrations, however, this period was too short to examine cardiometabolic outcomes [46]. These data suggest that bilirubin may induce molecular signaling pathways in adipose tissues, therefore, regulate adiposity and glucose sensitivity [47]. Furthermore, whether, circulating bilirubin is dependent upon weight-loss (e.g. related to reduced free radical production or inflammation) or is responsible in part for weight loss remains to be determined [36].

Data from National Health and Nutrition Examination Survey (NHANES) suggested that bilirubin levels were related to 26% lower risk of MetS [48, 49]. Congruent with our results, inverse relationship between serum bilirubin levels and MetS components were also shown in cross-sectional studies involving 12,342 Korean adults [15], 1568 Polish adults [50], and cohort study of 565 Kazakhs [51]. Moreover, bilirubin has been speculated to be a potential pre-disease biomarker for the development of MetS in asymptomatic Slovenian individuals [13], however, in comparison to our study, those studies had smaller sample size. All these clinical findings fit well with other studies demonstrated that serum bilirubin is involved in the lipid metabolism [52], regulation of PPARs [49, 53], or inhibition of inflammatory process [49, 54]. Additionally, a protective role of HMOX on glucose metabolism and insulin sensitivity should be considered [18, 55].

The major strengths of this study are its large sample size that allowed precise sex-stratified analyses and adjustment for a range of confounding factors. Also due to combining a set of correlated variables into a subset of principal components that explained most of the variation, the concern of multicollinearity was minimized [31]. For example, bilirubin was weakly inversely related with Lp(a) levels among men, while PC3_lipids_—an Lp(a) driven profile—showed a trend in the opposite direction indicating confounding by other highly correlated blood lipids in single-trait analyses. Furthermore, additional phenotype information might be captured by integrating multiple traits. This assertion is supported by the finding of Ried et al., who identified two novel loci for PC1_anthropometry_ that were not identified before in larger GWAS analyses for BMI, WHR adjusted for BMI, and height [29].

Our study has some limitations. First, the participants of UKB are mainly of European descent, so, whether the observed association could be applied to other ethnic groups and areas needs further investigation. Second, selection bias is of concern, which could lead to spurious associations in a situation where for example health conscious individuals are more likely to participate [56]. Although it is plausible that health consciousness is correlated with adiposity and/or blood lipid levels, it is less likely that such a correlation exists for bilirubin levels, which are mainly determined by genetic factors. Third, using PCA over datasets lead to transforming actual features in PCs, therefore PCs are difficult to interpret as compared to actual features. Lastly, the cross-sectional and observational nature of the study precludes conclusions about causality and although we controlled for a range of confounders, residual confounding cannot be excluded.

## Conclusions

In this cross-sectional analysis among men and women in the UKB, higher circulating levels of bilirubin were inversely associated with anthropometric indicators of adiposity. Inverse associations were also observed with systolic BP, TG levels and other unfavorable blood lipid fractions including apoB, while positive associations were observed with HDL-C and apoA-I. These findings suggest that routinely measured bilirubin could be indicative of cardiometabolic health. Moreover, if causality can be established in future studies, bilirubin could be a potential target to reduce risk of cardiometabolic diseases.

## Supplementary Information

**Additional file 1: Table S1A**. Principal components (PC) loading matrix (correlations) and explained variances for anthropometric variables in the UK Biobank.**Table S1B.** Principal components (PC) loading matrix (correlations) and explained variances for lipid variables in the UK Biobank.Figure S1. Mean values of anthropometric data of the top 5% of the study population.**Table S2A.** Association between bilirubin levels and principal components of anthropometric data and different sensitivity analyses in the UK Biobank.**Table S2B. **Association between bilirubin levels and principal components of lipid data and different sensitivity analyses in the UK Biobank.

## Data Availability

The UK Biobank resource is available to bona fide researchers for health-related research in the public interest. All researchers who wish to access the research resource must register with UK Biobank by completing the registration form in the Access Management System (AMS- https://bbams.ndph.ox.ac.uk/ams/). The datasets used and/or analyzed during the current study are available from the corresponding author on reasonable request.

## Abbreviations

ALT: Alanine transaminase
ALP: Alkaline phosphatase
AST: Aspartate transaminase
ApoA-I: Apolipoprotein A-I
ApoB: Apolipoprotein B
BIA: Bioelectrical impedance analysis
BMI: Body mass index
CVD: Cardiovascular disease
CRP: C-reactive protein
DM: Diabetes mellitus
FM: Fat mass
GGT: Gamma-glutamyl transpeptidase
HC: Hip circumference
HDL-C: High-density lipoprotein-cholesterol
HMOX: Heme oxygenase
LDL-C: Low-density lipoprotein-cholesterol
LDL/HDL: Low-density lipoprotein to high-density lipoprotein ratio
Lp(a): Lipoprotein (a)
MetS: Metabolic syndrome
PCA: Principal component analysis
PC: Principal component
TC: Total cholesterol
TC/HDL: Total cholesterol to high-density lipoprotein ratio
TG: Triglycerides
UGT1A1: Uridine-diphosphoglucuronate glucuronosyltransferase1A1
UKB: UK Biobank
WC: Waist circumference
WHR: Waist-hip ratio
