# Supplementary material for: Bilirubin as an indicator of cardiometabolic health: a cross-sectional analysis in the UK Biobank
Source: Cardiovasc Diabetol. 2022 Apr 18;21:54. doi: 10.1186/s12933-022-01484-x (PMC9017025; doi:10.1186/s12933-022-01484-x)
Supplement: Supplementary file 1 — Additional file 1: Table S1A. Principal components (PC) loading matrix (correlations) and explained variances for anthropometric variables in the UK Biobank.Table S1B. Principal components (PC) loading matrix (correlations) and explained variances for lipid variables in the UK Biobank.Figure S1. Mean values of anthropometric data of the top 5% of the study population.Table S2A. Association between bilirubin levels and principal components of anthropometric data and different sensitivity analyses in the UK Biobank.Table S2B. Association between bilirubin levels and principal components of lipid data and different sensitivity analyses in the UK Biobank. [file 12933_2022_1484_MOESM1_ESM.pdf]

# Bilirubin as an Indicator of Cardiometabolic Health: A Cross-Sectional Analysis in the UK Biobank

Nazlisadat Seyed Khoei[1], Karl-Heinz Wagner[1], Anja M. Sedlmeier[2], Marc J. Gunter[3], Neil Murphy[3], Heinz Freisling [3]*

*Correspondence:

Heinz Freisling (HF), Nutrition and Metabolism Branch, International Agency for Research on Cancer (IARC-WHO), 150 Cours Albert Thomas, 69372 Lyon CEDEX 08, France.

E-mail: Freislingh@iarc.fr

**Table S1A.** Principal components (PC) loading matrix (correlations) and explained variances for anthropometric variables in the UK Biobank.

**Table S1B.** Principal components (PC) loading matrix (correlations) and explained variances for lipid variables in the UK Biobank.

**Figure S1.** Mean values of anthropometric data of the top 5% of the study population.

**Table S2A.** Association between bilirubin levels and principal components of anthropometric data and different sensitivity analyses in the UK Biobank.

**Table S2B.** Association between bilirubin levels and principal components of lipid data and different sensitivity analyses in the UK Biobank.

**Table S1A**. Principal components (PC) loading matrix (correlations) and explained variances for anthropometric measures in the UK Biobank

| **Variables** | **PC 1** | **PC 2** | **PC 3** | **PC 4** |
|---|---|---|---|---|
| Height | 0.0786 | 0.8465 | 0.4303 | -0.0281 |
| Weight | 0.4778 | 0.2282 | -0.0799 | -0.4803 |
| BMI | 0.4720 | -0.1489 | -0.2842 | -0.4981 |
| WC | 0.4873 | -0.1178 | 0.1688 | 0.4007 |
| HC | 0.4487 | -0.1824 | -0.4016 | 0.5999 |
| WHR | 0.3223 | -0.4025 | 0.7334 | 0.0073 |
| **Eigen value** | 3.98 | 1.17 | 0.74 | 0.12 |
| **%** | 66.26 | 19.50 | 12.31 | 1.93 |
| **Cumulative** | 66.26 | 85.76 | 98.07 | 100 |

**Table S1B**. Principal components (PC) loading matrix (correlations) and explained variances for lipid concentrations in the UK Biobank

| **Variables** | **PC 1** | **PC 2** | **PC 3** | **PC 4** |
|---|---|---|---|---|
| ApoA-I | -0.1983 | 0.5088 | -0.0910 | 0.2848 |
| ApoB | 0.3697 | 0.2858 | 0.0137 | -0.1192 |
| ApoB/apoA-I | 0.4110 | -0.0481 | 0.0615 | -0.2546 |
| Lp (a) | 0.0278 | 0.0427 | 0.9626 | 0.2660 |
| TC | 0.2831 | 0.4539 | -0.0427 | 0.0389 |
| LDL-C | 0.3438 | 0.3569 | 0.0011 | -0.1015 |
| HDL-C | -0.2100 | 0.5302 | -0.0137 | -0.0227 |
| TG | 0.2558 | -0.1201 | -0.2420 | 0.8662 |
| TC/HDL | 0.4150 | -0.1245 | -0.0213 | 0.0514 |
| LDL/HDL | 0.4176 | -0.1045 | 0.0107 | -0.0624 |
| **Eigen value** | 5.46 | 2.63 | 1.01 | 0.71 |
| **%** | 54.61 | 26.33 | 10.15 | 7.15 |
| **Cumulative** | 54.61 | 80.94 | 91.09 | 98.23 |

Abbreviation: PC: principal components, BMI: body mass index, WC: waist circumference, HC: hip circumference, WHR: waist-hip ratio, ApoA-I: apolipoprotein A-I, ApoB: apolipoprotein B, Lp (a): lipoprotein (a), TC: total cholesterol, LDL-C: low-density lipoprotein cholesterol, HDL-C: high-density lipoprotein cholesterol, TG: triglycerides.

principal component analysis (PCA) is applied to in turn six anthropometric traits (i.e. height, weight, BMI, WC, HC, and WHR), and to ten lipid measures (apoA-I, apoB, apoB/apoA-I, Lp (a), TC, LDL-C, HDL-C, TG, LDL/HDL, and TC/HDL) combined for men and women. For each group of outcomes, the first four PC are extracted, which captured most of the variation of the input variables. PCA was performed on the standardized residuals of the anthropometric traits adjusted for age at recruitment, study center, and sex. The same analyses were repeated for lipid profiles. Those first four PCs or patterns combined in men and women with higher eigenvalues were considered as main contributors to each PC. The degree of correlation between variables and PCs are given by variable loadings. Therefore, higher component loading indicates higher influence of a given variable on a PC, which biological interpretation

**Figure S1.** Mean values of anthropometric data of the top 5% of the study population

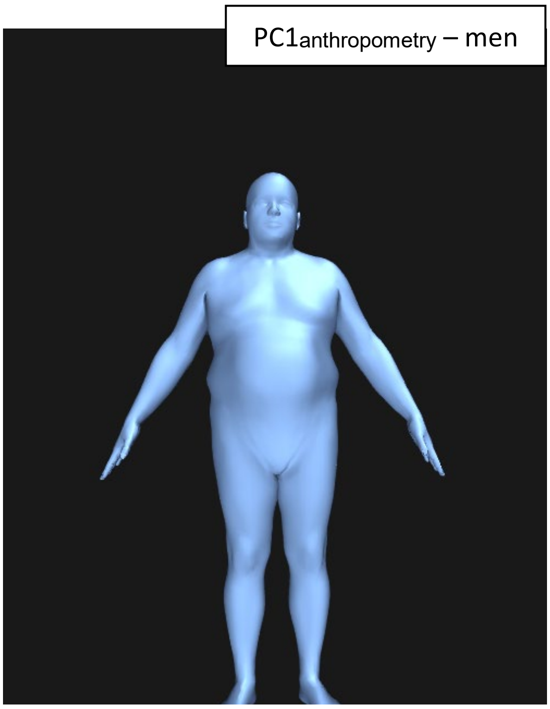


Height: 178cm; Weight: 121kg; Waist: 124cm; Hip: 121cm

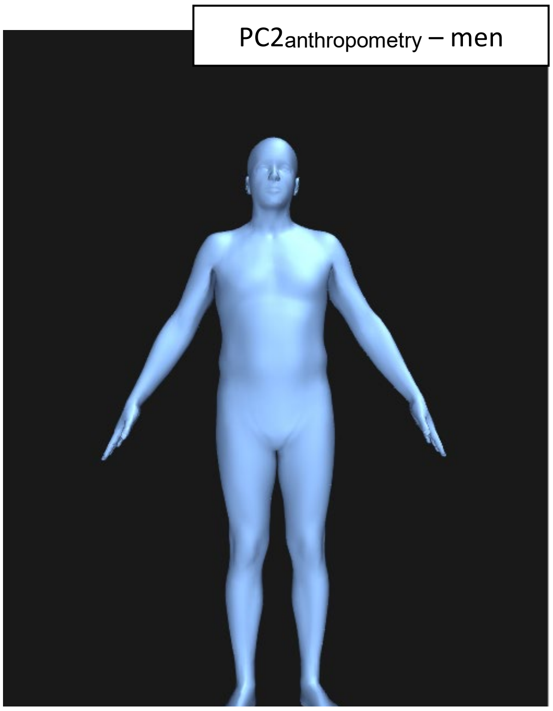


Height: 189cm; Weight: 95kg; Waist: 96cm; Hip: 108cm

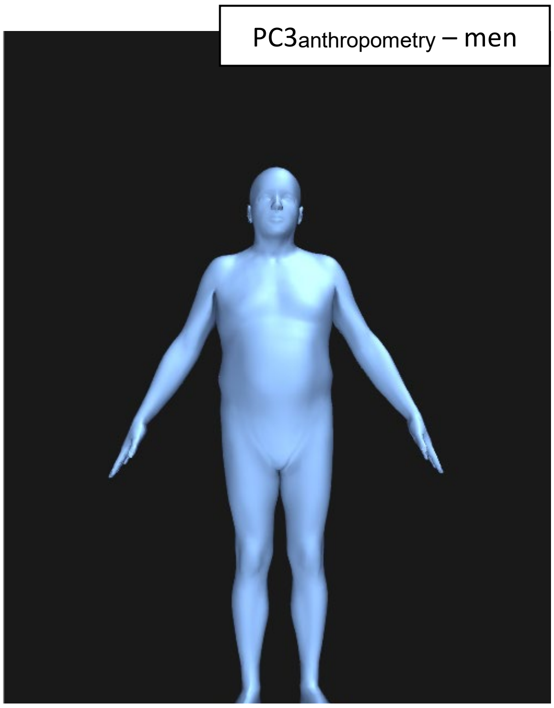


Height: 181cm; Weight: 93kg; Waist: 107cm; Hip: 102cm

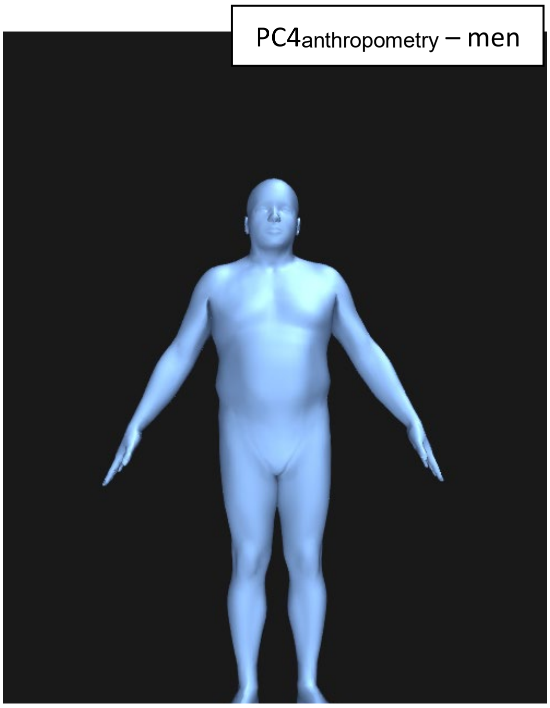


Height: 177cm; Weight: 102kg; Waist: 102cm; Hip: 104cm

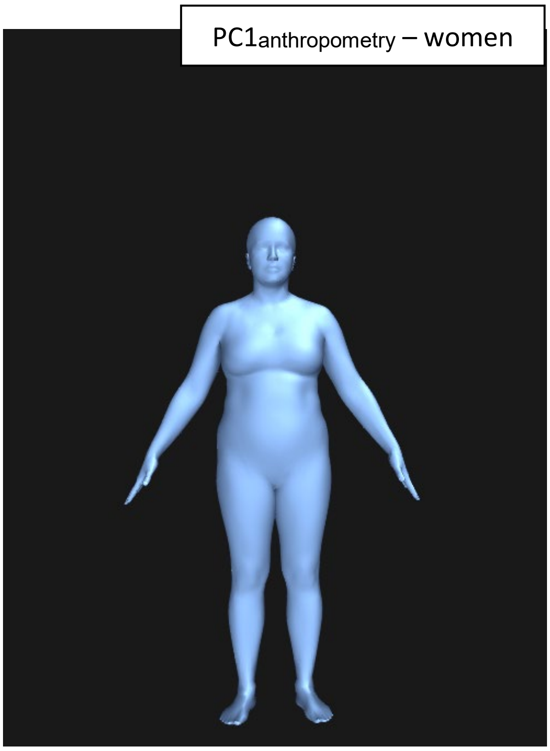


Height: 162cm; Weight: 72kg; Waist: 85cm; Hip: 103cm

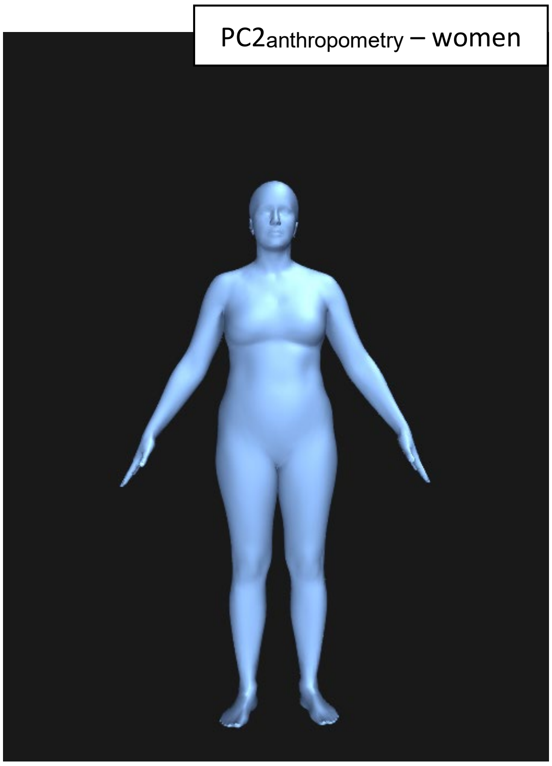


Height: 174cm; Weight: 78kg; Waist: 82cm; Hip: 107cm

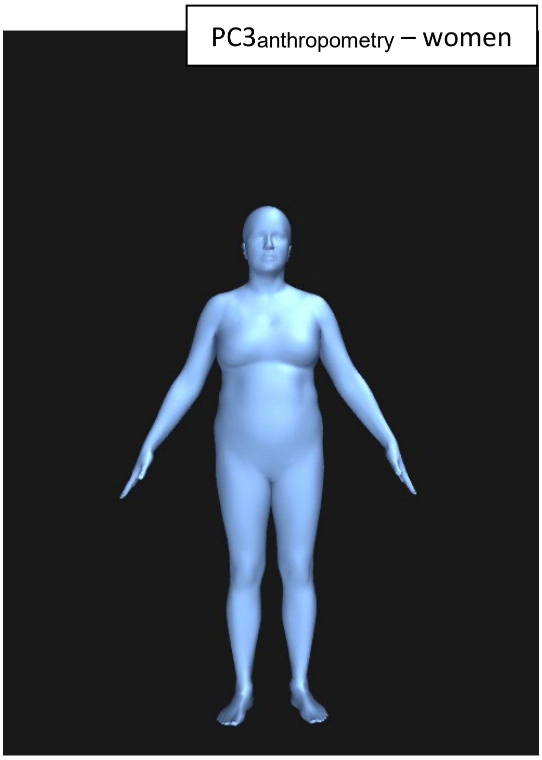


Height: 166cm; Weight: 72kg; Waist: 93cm; Hip: 99cm

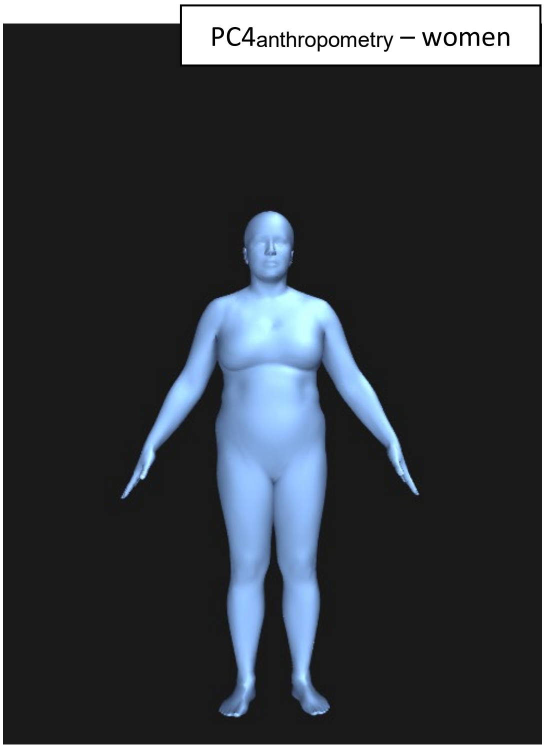


Height: 162cm; Weight: 79kg; Waist: 83cm; Hip: 101cm

**Table S2A.** Association between bilirubin levels and principal components of anthropometric data (regression standardized beta coefficients) and different sensitivity analyses in the UK Biobank

| Variable | Standardized beta coefficients | 99% CI | Adj R2 | Standardized beta coefficients | 99% CI | Adj R2 |
|---|---|---|---|---|---|---|
| | Men | | | Women | | |
| **PC1anthropometry: General adiposity** | | | | | | |
| Crude model (n=212,969/252,558) | -0.053 | -0.058 to -0.048 | 0.0035 | -0.141 | -0.146 to -0.135 | 0.0168 |
| Adj model (n=212,619/232,037) | -0.059 | -0.064 to -0.055 | 0.1562 | -0.124 | -0.130 to -0.119 | 0.1581 |
| Model S1 (n=194,498/223,449) | -0.059 | -0.064 to -0.054 | 0.1598 | -0.125 | -0.130 to -0.119 | 0.1566 |
| Model S2 (n=197,901/222,785) | -0.056 | -0.061 to -0.051 | 0.1415 | -0.121 | -0.126 to -0.115 | 0.1362 |
| Model S3 (n= 212,426/231,879) | -0.059 | -0.064 to -0.054 | 0.1573 | -0.124 | -0.129 to -0.119 | 0.1583 |
| Model S4 (n= 212,187/231,589) | -0.052 | -0.057 to –0.048 | 0.1678 | -0.096 | -0.102 to –0.091 | 0.2155 |
| **PC2anthropometry: Tall with low waist-hip ratio** | | | | | | |
| Crude model | 0.102 | 0.097 to 0.108 | 0.0109 | 0.110 | 0.105 to 0.115 | 0.0117 |
| Adjusted model | 0.088 | 0.083 to 0.094 | 0.0779 | 0.095 | 0.090 to 0.101 | 0.0773 |
| Model S1 | 0.089 | 0.083 to 0.094 | 0.0737 | 0.096 | 0.090 to 0.101 | 0.0742 |
| Model S2 | 0.087 | 0.083 to 0.093 | 0.0731 | 0.095 | 0.091 to 0.100 | 0.0706 |
| Model S3 | 0.089 | 0.083 to 0.094 | 0.0781 | 0.095 | 0.090 to 0.101 | 0.0773 |
| Model S4 | 0.085 | 0.08 to 0.091 | 0.0799 | 0.091 | 0.085 to 0.096 | 0.0791 |
| **PC3anthropometry: Tall with high waist-hip ratio** | | | | | | |
| Crude model | -0.015 | -0.019 to -0.010 | 0.0003 | 0.016 | 0.011 to 0.022 | 0.0002 |
| Adjusted model | -0.005 | -0.010 to -0.001 | 0.0197 | 0.013 | 0.007 to 0.019 | 0.0233 |
| Model S1 | -0.006 | -0.010 to -0.001 | 0.0204 | 0.014 | 0.007 to 0.020 | 0.0232 |
| Model S2 | -0.005 | -0.010 to -0.0004 | 0.0205 | 0.014 | 0.008 to 0.020 | 0.0237 |
| Model S3 | -0.005 | -0.010 to -0.0008 | 0.0197 | 0.013 | 0.007 to 0.019 | 0.0233 |
| Model S4 | -0.005 | -0.010 to -0.0004 | 0.0198 | 0.006 | 0.000 to 0.012 | 0.0270 |
| **PC4anthropometry: High BMI and weight, with relatively small hip and waist circumference** | | | | | | |
| Crude model | -0.010 | -0.016 to -0.005 | 0.0001 | -0.032 | -0.037 to -0.026 | 0.0010 |
| Adjusted model | -0.002 | -0.007 to -0.004 | 0.0254 | -0.028 | -0.034 to -0.023 | 0.0062 |
| Model S1 | -0.0003 | -0.006 to 0.005 | 0.0261 | -0.027 | -0.033 to -0.022 | 0.0060 |
| Model S2 | -0.001 | -0.007 to -0.005 | 0.0243 | -0.026 | -0.032 to -0.021 | 0.0058 |
| Model S3 | -0.002 | -0.007 to -0.004 | 0.0256 | -0.028 | -0.034 to -0.023 | 0.0062 |
| Model S4 | -0.001 | -0.007 to -0.004 | 0.0254 | -0.025 | -0.031 to -0.020 | 0.0070 |

**Table S2B.** Association between bilirubin levels and principal components of lipid data (regression standardized beta coefficients) and different sensitivity analyses in the UK Biobank

| Variable | Standardized beta coefficients | 99% CI | Adj R2 | Standardized beta coefficients | 99% CI | Adj R2 |
|---|---|---|---|---|---|---|
| | Men | | | Women | | |
| **PC1lipids: Dyslipidemia** | | | | | | |
| Crude model (n=156,113/182,253) | -0.090 | -0.097 to -0.084 | 0.0080 | -0.109 | -0.115 to -0.103 | 0.0120 |
| Adj model (n=155,922/167,745) | -0.085 | -0.091 to -0.079 | 0.1697 | -0.081 | -0.087 to -0.076 | 0.1406 |
| Model S1 (n=143,241/162,054) | -0.090 | -0.096 to -0.084 | 0.1483 | -0.086 | -0.092 to -0.080 | 0.1409 |
| Model S2 (n=145,557/161,340) | -0.095 | -0.101 to -0.089 | 0.1588 | -0.088 | -0.094 to -0.082 | 0.1403 |
| Model S3 (n= 155,837/167,745) | -0.090 | -0.096 to -0.084 | 0.1763 | -0.086 | -0.092 to -0.080 | 0.1434 |
| **PC2lipids: Anti-atherogenic** | | | | | | |
| Crude model | 0.061 | 0.054 to 0.068 | 0.0034 | 0.091 | 0.085 to 0.097 | 0.0088 |
| Adjusted model | 0.043 | 0.037 to 0.049 | 0.2264 | 0.071 | 0.065 to 0.076 | 0.1926 |
| Model S1 | 0.050 | 0.045 to 0.057 | 0.1806 | 0.078 | 0.072 to 0.084 | 0.1760 |
| Model S2 | 0.045 | 0.039 to 0.052 | 0.1792 | 0.074 | 0.068 to 0.080 | 0.1613 |
| Model S3 | 0.047 | 0.041 to 0.053 | 0.2224 | 0.075 | 0.070 to 0.081 | 0.1922 |
| **PC3lipids: High levels of lipoprotein (a)** | | | | | | |
| Crude model | 0.027 | 0.021 to 0.034 | 0.0007 | 0.026 | 0.020 to 0.032 | 0.0007 |

| | | | | | | |
|---|---|---|---|---|---|---|
| Adjusted model | 0.033 | 0.026 to 0.035 | 0.0133 | 0.026 | 0.020 to 0.032 | 0.0080 |
| Model S1 | 0.024 | 0.017 to 0.031 | 0.0102 | 0.018 | 0.011 to 0.024 | 0.0069 |
| Model S2 | 0.026 | 0.019 to 0.033 | 0.0114 | 0.018 | 0.011 to 0.024 | 0.0066 |
| Model S3 | 0.026 | 0.019 to 0.033 | 0.0115 | 0.018 | 0.012 to 0.025 | 0.0068 |
| **PC4**$_{lipids}$: **High levels of triglycerides** | | | | | | |
| Crude model | 0.033 | 0.025 to 0.041 | 0.0004 | 0.031 | 0.024 to 0.038 | 0.0007 |
| Adjusted model | 0.039 | 0.031 to 0.047 | 0.0133 | 0.031 | 0.023 to 0.038 | 0.0080 |
| Model S1 | 0.029 | 0.021 to 0.038 | 0.0102 | 0.021 | 0.013 to 0.029 | 0.0051 |
| Model S2 | 0.032 | 0.023 to 0.040 | 0.0114 | 0.022 | 0.014 to 0.030 | 0.0066 |
| Model S3 | 0.032 | 0.024 to 0.040 | 0.0115 | 0.023 | 0.015 to 0.031 | 0.0068 |

Abbreviation: PC: principal components, CI: confidence interval, Adj: adjusted.
Multivariable-adjusted linear regression analyses were used to estimate the cross-sectional association between 1 SD increase in log-transformed bilirubin levels and separately each cardiometabolic health indicator (for 1 SD changes in PC scores) adjusted for age at recruitment, ethnicity, alcohol consumption, alcohol consumption frequency, smoking status, physical activity, liver enzyme (alanine transaminase, ALT), chronic diseases (heart problems and diabetes), medications (for cholesterol, blood pressure, diabetes, or exogenous hormones), qualifications, and ever use of hormones in women.
Model S1: Excluding participants with CVD (heart attack, angina, and stroke) at baseline
Model S2: Excluding participants with diabetes at baseline
Model S3: Excluding participants with liver enzymes beyond 90th percentile (including alanine transaminase (ALT), aspartate transaminase (AST), alkaline phosphatase (ALP), and gamma-glutamyl transpeptidase (GGT).
Model S4: Linear regression analyses were additionally adjusted with CRP.
N.B.: PC4$_{anthropometry}$, PC3$_{lipids}$, and PC4$_{lipids}$ showed strong non-linear associations (see main findings Figures 2A and 2B) and are shown here for completeness.
